# The Anti-Inflammatory Activity of a Novel Fused-Cyclopentenone Phosphonate and Its Potential in the Local Treatment of Experimental Colitis

**DOI:** 10.1155/2015/939483

**Published:** 2015-04-09

**Authors:** Dorit Moradov, Helena Shifrin, Efrat Harel, Mirela Nadler-Milbauer, Marta Weinstock, Morris Srebnik, Abraham Rubinstein

**Affiliations:** ^1^Faculty of Medicine, School of Pharmacy Institute for Drug Research, The Hebrew University of Jerusalem, P.O. Box 12065, 91120 Jerusalem, Israel; ^2^The Harvey M. Krueger Family Center for Nanoscience and Nanotechnology, The Hebrew University, 91904 Jerusalem, Israel; ^3^The David R. Bloom Center of Pharmacy, The Hebrew University, 91120 Jerusalem, Israel

## Abstract

A novel fused-cyclopentenone phosphonate compound, namely, diethyl 3-nonyl-5-oxo-3,5,6,6a-tetrahydro-1*H*-cyclopenta[*c*]furan-4-ylphosphonate (P-5), was prepared and tested *in vitro* (LPS-activated macrophages) for its cytotoxicity and anti-inflammatory activity and *in vivo* (DNBS induced rat model) for its potential to ameliorate induced colitis. Specifically, the competence of P-5 to reduce TNF*α*, IL-6, INF*γ*, MCP-1, IL-1*α*, MIP-1*α*, and RANTES in LPS-activated macrophages was measured. Experimental colitis was quantified in the rat model, macroscopically and by measuring the activity of tissue MPO and iNOS and levels of TNF*α* and IL-1*β*. It was found that P-5 decreased the levels of TNF*α* and the tested proinflammatory cytokines and chemokines in LPS-activated macrophages. In the colitis-induced rat model, P-5 was effective locally in reducing mucosal inflammation. This activity was equal to the activity of local treatment with 5-aminosalicylic acid. It is speculated that P-5 may be used for the local treatment of IBD (e.g., with the aid of colon-specific drug platforms). Its mode of action involves inhibition of the phosphorylation of MAPK ERK but not of p38 and had no effect on I*κ*B*α*.

## 1. Introduction

Inflammatory bowel disease (IBD), a chronic inflammation of the gut, is characterized by a profound infiltration of macrophages and a continuous efflux of proinflammatory cytokines [1, 2] into the intestinal mucosa. The typical overproduction of tumor necrosis factor alpha (TNF*α*) in IBD has already led to the use of anti-TNF*α* monoclonal antibodies (e.g., infliximab) to block the access of TNF*α* to its receptor for the specific treatment of IBD [3, 4]. However, the use of these drugs is associated with severe adverse effects such as immunogenicity [5, 6], risk of lymphoma, and neuropathy [7, 8]. Thus, a rational approach to TNF*α*-based therapy may use inhibitors (e.g., metalloproteinase inhibitors) [9, 10] to prevent the TNF*α*-converting enzyme- (TACE-) mediated release of the soluble form of TNF*α* from its cell membrane-bound precursor into the vicinity of the inflamed regions [11, 12].

Phosphonate compounds possess a variety of pharmacological properties including antibacterial activity [13, 14] and nucleic acid or amino acid mimetic properties [15]. Phosphonate-phospholipid analogues can inhibit proinflammatory lipid mediators such as phospholipase A2 [16]. Phosphonate compounds are also efficient serine protease inhibitors [17]. They possess chelating properties [18] that enable them to inhibit the activity of zinc dependent enzymes such as matrix metalloproteinase and TACE [19–21]. They were also shown to ameliorate inflammation by reducing the activity of reactive oxygen species, as well as decreasing the levels of secreted TNF*α* [22–24].

Based on a synthetic method previously described by us [25], the goals of the present study were to (a) prepare the novel fused-cyclopentenone phosphonate, diethyl 3-nonyl-5-oxo-3,5,6,6a-tetrahydro-1*H*-cyclopenta[*c*]furan-4-ylphosphonate (denoted by P-5), (b) test whether P-5 can inhibit local secretion of TNF*α*, (c) examine,* in vitro* and* in vivo*, whether P-5 can ameliorate chronic inflammation, such as experimental colitis, and (d) explore mechanistically the possible anti-inflammatory effect of P-5.

## 2. Materials and Methods

### 2.1. Materials

Unless stated otherwise, all materials were purchased from Sigma (St. Louis, MO, Germany). Dimethyl sulfoxide (DMSO) was purchased from Merck (Darmstadt, Germany). Solvents were of analytical grade. Water was filtered and deionized by reverse osmosis (Barnstead Nanopure, Waltham, MA, USA). Thioglycollate broth was purchased from Difco (Lawrence KS, USA). Recombinant human TACE and the peptide Mca-P-L-A-Q-A-V-Dpa-R-S-S-S-R-NH_2_ (Fluorogenic Peptide Substrate III) were purchased from R&D Systems, MN, USA. RIPA lysis buffer, containing a cocktail of phosphatases and proteases inhibitor (1%), was purchased from Bet Haemek, Israel.

Antibodies against I*κ*B*α*, ERK, phosphorylated ERK (p-ERK), p38, phosphorylated p38 (p-p38), tubulin, iNOS, and GAPDH were purchased from Santa Cruz Biotechnologies, Santa Cruz, CA, USA. IRDye conjugated fluorescent secondary antibodies were as follows: donkey anti-mouse 680 was purchased from Rockland Immunochemicals, PA, USA; Alexa Fluor goat anti-rabbit 488 was purchased from Molecular Probes, NY, USA. TNF*α* and IL-6 ELISA kits were purchased from R&D Systems, MN, USA. IL-1*β* ELISA kit was purchased from PeproTech, Rocky Hill, USA. The IL-1*α*, INF*γ*, MCP-1, MIP-1*α*, and RANTES ELISA kit were purchased from Quansys biosciences, Utah, USA.

### 2.2. P-5 Preparation

**Scheme 1:**
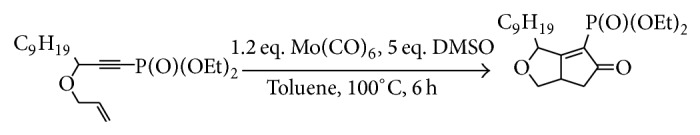
Synthesis of diethyl 3-nonyl-5-oxo-3,5,6,6a-tetrahydro-1*H*-cyclopenta[*c*]furan-4-ylphosphonate (P-5).

Diethyl 3-nonyl-5-oxo-3,5,6,6a-tetrahydro-1*H*-cyclopenta[*c*]furan-4-ylphosphonate (P-5, Scheme 1) was synthesized by Pauson-Khand reaction from diethyl 3-(allyloxy)dodec-1-ynylphosphonate using Mo(CO)_6_ and DMSO as described previously [25]. Briefly, to Mo(CO)_6_ (1.2 eq) in dry toluene diethyl 3-(allyloxy)dodec-1-ynylphosphonate (1 eq) was added, followed by the addition of DMSO (5 eq). After refluxing for 6 h at 100°C the reaction mixture was cooled and ethyl acetate was added. The entire mixture was filtered through silica gel and the product was separated on silica gel column using gradient eluent of methanol/dichloromethane. The reaction yield was 58%.

### 2.3. Animals, Maintenance, and Euthanasia

C57BL/6 female mice (6–8 weeks old) and Sabra male rats (200–250 g), obtained from Harlan Laboratories, Jerusalem, Israel, were kept under constant environmental conditions (22°C, 12 h light/dark cycles) and fed with standard laboratory chow and tap water. All animal studies were conducted in accordance with the Principles of Laboratory Animal Care (NIH publication number 85-23, revised 1985). The joint Ethics Committee (IACUC) of the Hebrew University and Hadassah Medical Center approved the study protocol for animal welfare. The Hebrew University Animal Facility is an AAALAC international accredited institute (number 1285). Sedation of the mice was performed by isoflurane (USP, Terrell, Minrad Inc., USA). Euthanasia of the sedated mice was performed by cervical dislocation. Sedation of the rats was performed by intraperitoneal injection of a mixture of 100 mg/kg rat body weight of Ketamine (Ketaset, Fort Dodge, USA) and 2 mg per rat of Xylazine (Sedaxylan, Nederland). Euthanasia of the sedated rats was performed by puncture of the chest wall.

### 2.4. Peritoneal Macrophages: Induction and Harvesting

The mice were injected intraperitoneally with 1.5 mL of a 3% thioglycollate broth and sacrificed 4 days later. Immediately after the euthanasia, the recruited macrophages were aspirated from the inflamed tissue. The aspirated liquid containing cells was centrifuged; the suspended cells were plated in a 96-microwell flat-bottom plate (NUNC, Denmark) at a concentration of 1.5 × 10^5^ cells/well. After incubation, the medium was aspirated and the cells were rinsed with PBS to remove nonadherent cells.

### 2.5. P-5 Activity, Cytotoxicity, and TACE Inhibition Assessments

P-5 activity was tested towards activated macrophages. Elevated concentrations (1–20 *μ*M) of P-5 in absolute ethanol, diluted with DMEM, were added to each well of a 96-microwell plate preseeded with the peritoneal macrophage cells (3–5 wells for each study). The macrophages were then activated by the addition of 25 *μ*L of lipopolysaccharide (LPS, from* Escherichia coli*, serotype 0111:B4, Sigma Ltd.) to each well (final concentration of 1 *μ*g/mL). The activated macrophages were then incubated and the supernatant fluid of each well was collected and kept frozen (−80°C) until analysis of TNF*α*. The steroid drug, budesonide, was used as a positive control at a final concentration of 10 *μ*M. Wells containing LPS-activated cells without the addition of P-5 or budesonide served as negative (untreated) controls.

P-5 cytotoxicity was assessed by the MTT test. The 50% lethal concentration (LC_50_) of P-5 was measured at a concentration range of 1–40 *μ*M.

The possible inhibitory effect of P-5 on recombinant human TACE was assessed by incubating the enzyme (0.1 ng/mL) with increasing concentrations (1, 10 or 50 *μ*M) of the compound in the presence of the fluorescent peptide substrate Mca-P-L-A-Q-A-V-Dpa-R-S-S-S-R-NH_2_ [26]. The fluorescence which resulted by the TACE cleavage was measured at *λ*ex = 320 nm/*λ*em = 405 nm. Doxycycline (100 *μ*M) was used as a positive control in the inhibition study.

### 2.6. Cytokine Levels Determination

TNF*α* and IL-6 levels were measured by ELISA assay, employing a commercial kit (R&D Systems, MN, USA) according to the manufacturer's instructions. IL-1*α*, INF*γ*, MCP-1, MIP-1*α*, and RANTES levels were analyzed by Q-Plex arrays, a multiplex commercial ELISA kit (Quansys biosciences, Utah, USA) according to the manufacturer's instructions.

### 2.7. I*κ*B*α*, p38, and ERK Analysis

Macrophage levels of I*κ*B*α*, the protein kinase p38, and the extracellular signal-regulated kinase ERK were determined by Western blot. Isolated macrophages from the mice peritoneum were plated in 6-well culture plates (5 × 10^6^ cells/well). After 2-3 h, the incubation medium was aspirated and nonadherent cells were washed away with sterile PBS. P-5 (1 or 5 *μ*M in a fresh complete DMEM medium) was then added. The control wells contained fresh medium only. Two hours later, the macrophages were activated with 5 *μ*g/mL of LPS and further incubated (15 min) for I*κ*B*α* analysis, 30 min for mitogen-activated protein kinases (MAPKs) phosphorylated* p*38, and 30 min for phosphorylated ERK analysis. The cells were then harvested and centrifuged and the cell pellets were shaken for 30 min, on ice, in a RIPA lysis buffer containing a cocktail of phosphatase and proteases inhibitors (1%). Protein concentration in the supernatant was determined by bicinchoninic acid protein assay kit (Thermo Scientific, USA). Protein samples (20 *μ*g) were separated on 10% SDS polyacrylamide gels with 4.5% SDS stacking gel. Samples were electrotransferred onto nitrocellulose membranes (0.45 *μ*m; Schleicher, Dassel, Germany). Blots were probed with antibodies against I*κ*B*α* (1 : 500), ERK (1 : 300), phosphorylated ERK (p-ERK) (1 : 300), p38 (1 : 1000), phosphorylated p38 (p-p38) (1 : 600), and tubulin (1 : 1000). The nitrocellulose membranes were incubated with the appropriate primary antibodies and then incubated (1 h, room temperature) with appropriate IRDye conjugated fluorescent secondary antibodies: donkey anti-mouse 680 and Alexa Fluor goat anti-rabbit 488. IRDye conjugates are all optimized for the Odyssey Infrared Imaging System (LI-COR Biosciences, Lincoln, NE, USA). The densities of the obtained protein bands were quantified using TINA image analyzer software (version 2.07d; Raytest, Straubenhardt, Germany). The amount of I*κ*B*α* was quantified and normalized to tubulin. The amount of p-p38 and p-ERK was quantified and normalized to band density of the nonphosphorylated entities, respectively.

### 2.8. Induction of Experimental Colitis in Rats and Treatment Protocol

The rats were deprived of food with free access to water 24 h prior to the colitis induction which was performed under light sedation (isoflurane inhalation) by intracolonic administration of 30 mg of DNBS in 1 mL of ethanol 25% (v/v) [27, 28]. One hour after the colitis induction P-5, at a dose of 10 mg/kg body weight, was administered intracolonically in 0.5 mL PBS containing 5% Tween 80 and 5% of absolute ethanol. The administration was repeated every 12 hours over a period of 3 days. Rats dosed with a 5-aminosalicylic acid (5-ASA) enema (268 mg/kg body weight) served as a positive control group [29]. A group of healthy rats was used as a naive control group. A group of untreated DNBS-induced rats served as a nontreated control group. On the fourth day, the rats were sacrificed and their colons exteriorized through a longitudinal abdominal incision.

### 2.9. Quantification of Inflammation Severity

The distal 10 cm of each colon was removed, cut open, and rinsed with ice-cold PBS, pH 7.4. Colon sections were blotted dry and weighed and the length was measured. Ulcerated and inflamed regions were identified. Scoring of the ulcerated areas was conducted by assigning 0.5 points for each 5 mm of ulcerated tissue [30].

### 2.10. Tissue Analysis of Inflammatory Markers

Tissue activity of* myeloperoxidase* (MPO) was analyzed in tissue homogenates (Polytron, Kinematiea GmbH, Germany) in 0.02 M phosphate-buffer, pH 7.4. After centrifugation and resuspension of the pellet in ice-cold phosphate buffer (50 mM, pH 6.0) containing 0.5% of hexadecyltrimethylammonium bromide (to release MPO from the primary granules of the neutrophils), the suspension was freeze-thawed, sonicated, and centrifuged. 10 *μ*L of the supernatant was then added to 290 *μ*L of phosphate buffer, containing* o*-dianisidine hydrochloride and hydrogen peroxide (5 × 10^−4^% v/v). The kinetics of absorbance change was measured at 460 nm over 30 sec. MPO activity was calculated using a 6-point calibration curve employing purified peroxidase [31]. MPO activity (per *μ*g total tissue protein) was expressed as a fraction (in %) of the enzyme activity normalized to the tissue activity in the untreated control group.

TNF*α* and IL-1*β* levels in the homogenized colon tissues were measured using ELISA kits. After centrifugation, the separated supernatant was poured into a 96-well MaxiSorb ELISA plate and processed according to the manufacturer's protocol. Cytokine levels (pg range) were expressed as a fraction (in %) of cytokine concentration in the colon tissues and were normalized to both total tissue protein and cytokine level in the untreated control group.

Inducible nitric oxide synthase (iNOS) activity was determined by immunoblotting as described above with relevant modifications. Colon tissues were homogenized in RIPA lysis buffer. The samples were blotted with antibodies against NOS2 (1 : 500) and GAPDH (1 : 1000). Tissue amount of iNOS was normalized to GAPDH levels.

*Protein tissue levels* were measured by the Bradford method [32] to allow normalizing of MPO activity and levels of TNF*α* and IL-1*β* to total tissue protein (pg/*μ*g tissue).

### 2.11. Statistical Analysis

The results are expressed as means ± S.D. values. Differences between data obtained from cells treated with LPS and cells treated with LPS plus P-5 at various concentrations and differences between data obtained from DNBS-induced rats with and without P-5 or 5-ASA were analyzed by ANOVA using IBM SPSS Statistics Ver. 19, followed by Duncan's post hoc test. A *p* value of <0.05 was considered to be significant.

## 3. Results

**Figure 1:**
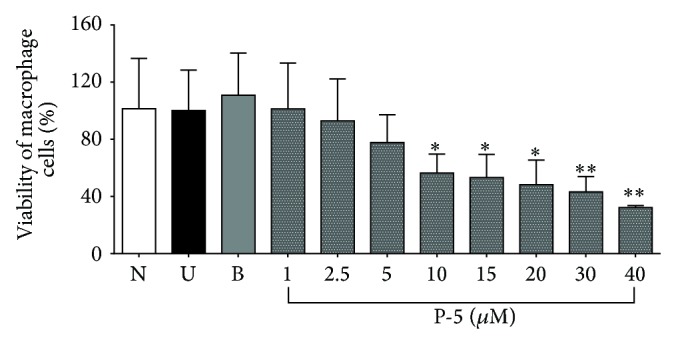
Relative cytotoxicity (expressed in % of the untreated cells) of low concentration range (1–40 *μ*M) of P-5 towards LPS-activated mouse peritoneal macrophages. N: naive control; U: untreated control; B: budesonide (10 *μ*M, positive control). Shown is the mean of three different experiments ± S.D. ANOVA of P-5 cytotoxicity was* F*_10,48_ = 5.32, *p* < 0.00001. ^∗^ *p* < 0.05; ^∗∗^ *p* < 0.01 compared to untreated macrophages.

**Figure 2:**
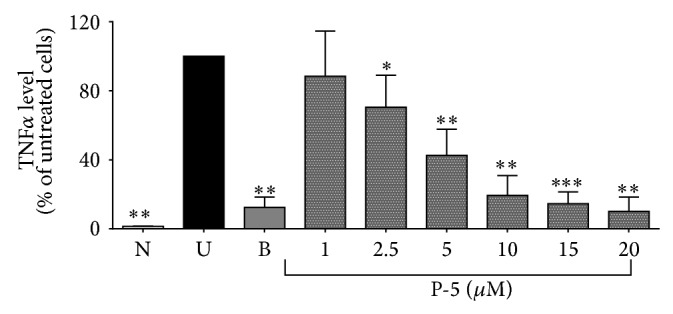
The reduction of TNF*α* levels (expressed as the fraction, in %, of TNF*α* level secreted by untreated cells) caused by a low concentration range (1–20 *μ*M) of P-5 as measured in LPS-activated macrophages. N: naive control; U: untreated control; B: budesonide (10 *μ*M, positive control). Shown is the mean of three different experiments ± S.D. ANOVA of the effect of P-5 on reduction of TNF*α* was* F*_8,97_ = 119.21, *p* < 0.00001. ^∗^ *p* < 0.05; ^∗∗^ *p* < 0.01; ^∗∗∗^ *p* < 0.001, compared to untreated macrophages.

The cytotoxicity of the fused-cyclopentenone phosphonate compound P-5 is shown in Figure 1, which demonstrates that its LC_50_ is 20 *μ*M and that, in concentration of 5 *μ*M or less, P-5 did not show any cytotoxicity towards the cells. The effect of P-5 on TNF*α* levels in the peritoneal macrophages was measured and its IC_50_ was identified as 6.1 *μ*M.  Figure 2 shows that 10 *μ*M of P-5 caused a reduction of 80% in TNF*α* secretion. At this dose, the viability of the cells was found to be 60% (Figure 1) which may be the cause for the profound reduction in TNF*α* levels. However, at a concentration of 5 *μ*M which showed no cytotoxicity toward the cells (Figure 1), there was a reduction of about 50% in TNF*α* levels (Figure 2).  Figure 2 also shows that P-5 reduced TNF*α* levels in the peritoneal macrophages in a dose-dependent manner.

**Figure 3:**
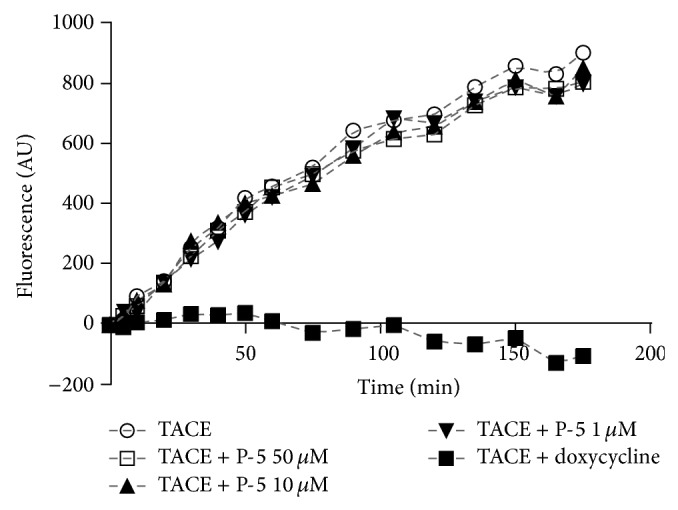
Cleavage of the fluorescent peptide substrate Mca-P-L-A-Q-A-V-Dpa-R-S-S-S-R-NH_2_ by purified human recombinant TACE in the presence of elevated concentrations of P-5 or doxycycline.

To elucidate whether the reduction in TNF*α* levels caused by P-5 was a result of TACE inhibition, increasing amounts of the compound were incubated with the fluorescently tagged TACE peptide substrate Mca-P-L-A-Q-A-V-Dpa-R-S-S-S-R-NH_2_ in the presence of purified human recombinant TACE. A similar study with 100 *μ*M of doxycycline served as a positive control. The results shown in Figure 3 demonstrate that TACE inhibition was not the cause for P-5 activity.

**Figure 4:**
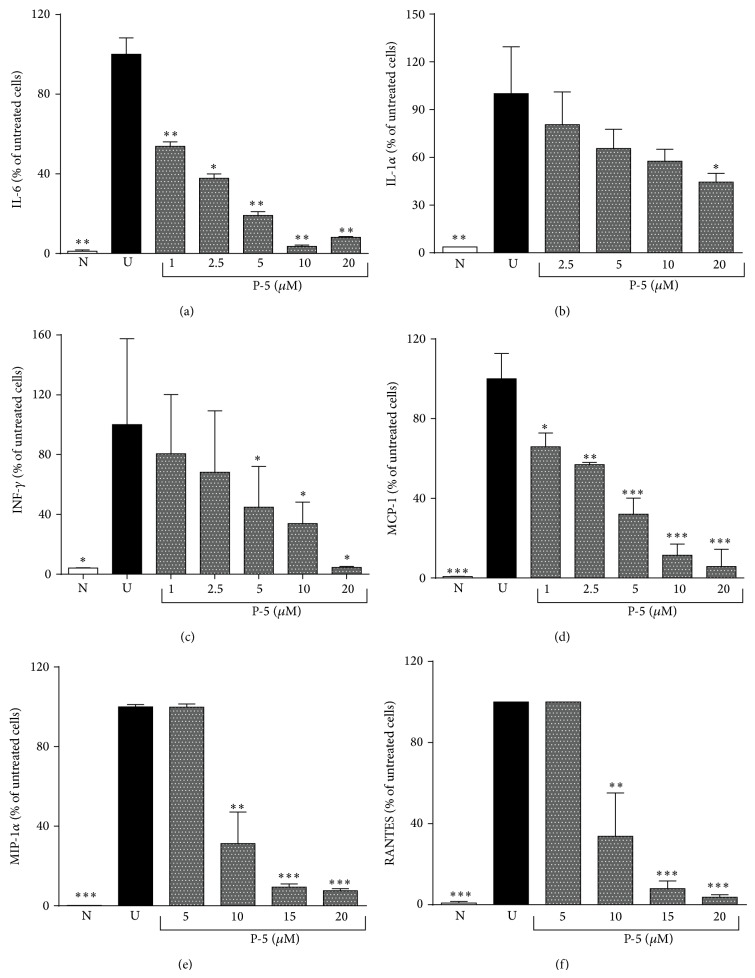
The attenuating effect of P-5 on the secretion of IL-6, IL-1*α*, INF*γ*, MCP-1, MIP-1*α*, and RANTES in LPS-activated macrophages. Results are expressed as the fraction (in %) of the cytokine level secreted by untreated activated cells. N: naive control; U: untreated control. Shown are the mean results ± S.D. (*n* = 3-4). ANOVA of the effect of P-5 at a series of concentrations on IL-6 levels was* F*_7,33_ = 115.36, *p* < 0.00001, on IL-1*α* was* F*_6,20_ = 8.06, *p* < 0.001, on INF*γ* was* F*_6,19_ = 4.64, *p* < 0.01, on MCP-1 was* F*_6,20_ = 73.57, *p* < 0.00001, on MIP-1*α* was* F*_7,23_ = 219.00, *p* < 0.00001, and on RANTES was* F*_5,17_ = 100.54, *p* < 0.00001. ^∗^ *p* < 0.05; ^∗∗^ *p* < 0.01; ^∗∗∗^ *p* < 0.001 compared to untreated cells.

**Figure 5:**
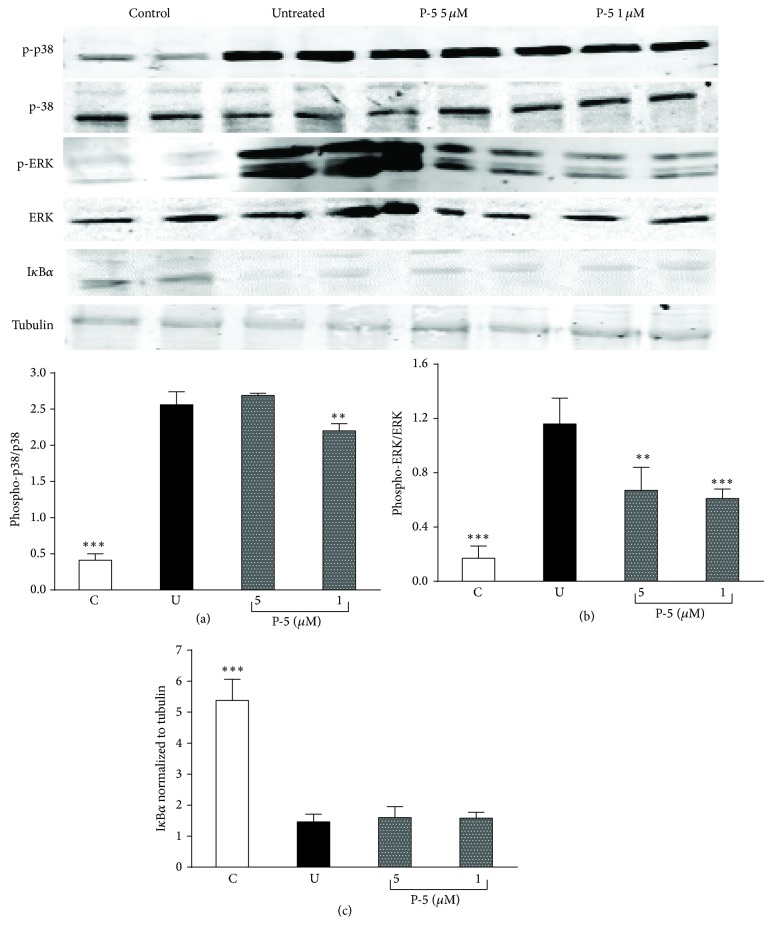
Western blot and densitometry analysis of p-p38 (normalized to p38; (a)), p-ERK (normalized to ERK; (b)), and I*κ*B*α* (normalized to tubulin; (c)) in activated macrophages lysates after incubation with 1 or 5 *μ*M of P-5. C: naive control; U: untreated control. Shown are the mean ± S.D results. of 4–6 measurements. ANOVA of the effect of 1 and 5 *μ*M of P-5 on the tested regulatory proteins were* F*_3,16_ = 353.02 *p* < 0.00001 for p-38,* F*_3,20_ = 47.19 *p* < 0.00001 for ERK, and* F*_3,16_ = 97.01 *p* < 0.00001 for I*κ*B*α*. ^∗∗^ *p* < 0.01; ^∗∗∗^ *p* < 0.001; compared to untreated cells.

Figure 4 shows how P-5 attenuated the secretion of a series of cytokines and chemokines in a dose dependent manner (dose range of 1–20 *μ*M) in the LPS-activated macrophages. While P-5 reduced IL-6, INF*γ*, and MCP-1 levels at its lowest concentration (1 *μ*M; Figures 4(a), 4(c), and 4(d)), higher doses were required for reducing the levels of IL-1*α* (2.5 *μ*M; Figure 4(b)), MIP-1*α*, and RANTES (10 *μ*M; Figures 4(e) and 4(f)). Since P-5 was not associated with TACE inhibition, its effect on p38, ERK, and I*κ*B*α* levels was also queried. The Western bolt analysis of p-p38, p-ERK, and I*κ*B*α* shows that while P-5 did not affect p38 and I*κ*B*α* levels, it significantly reduced the phosphorylation of ERK (Figure 5).

The local anti-inflammatory activity of P-5 as assessed, macroscopically, in the colon of DNBS-induced rats is summarized in Table 1, which shows that, after rectal administration of P-5, the weight of the inflamed colons was reduced by 33% (43% reduced by 5-ASA, the positive control). Ulceration scoring revealed that both P-5 and 5-ASA treatments reduced the severity of ulceration.

**Figure 6:**
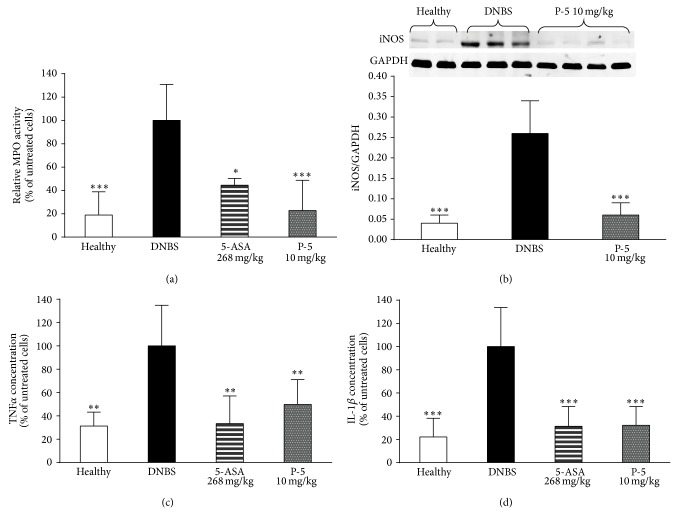
MPO activity (a), iNOS expression (b), TNF*α* (c), and IL-1*β* (d) tissue levels in the colons of healthy rats, untreated DNBS-induced rats, and DNBS-induced rats treated with P-5 and 5-ASA. Results are normalized to total tissue protein. (a), (c), and (d) are expressed as a fraction (in %) of the tissue biomarker levels in the DNBS-treated group. (b) is normalized to the house keeping gene, GAPDH. Shown are the mean ± S.D. values of 3–7 rats in each group. ANOVA of the effect of P-5 on MPO activity was* F*_3,19_ = 13.63 *p* < 0.0001, on iNOS expression was* F*_2,18_ = 37.57 *p* < 0.00001, on TNF*α* level was* F*_3,22_ = 9.00 *p* < 0.001, and on IL-1*β* level was* F*_3,31_ = 22.61 *p* < 0.00001. ^∗^ *p* < 0.05; ^∗∗^ *p* < 0.01; ^∗∗∗^ *p* < 0.001 compared with the DNBS group.

Quantification of the severity of inflammation was conducted by measuring tissue MPO activity, iNOS expression, and TNF*α* and IL-1*β* levels.  Figure 6 shows that MPO activity was increased by 80% in the colon tissues of the DNBS-induced rats, untreated group. Local treatment with P-5 attenuated the enzyme activity almost back to normal, while 5-ASA reduced the activity by 56%. P-5 reduced iNOS expression by 78% compared to the DNBS-induced group and decreased the levels of TNF*α* and IL-1*β* by 50 and 68%, respectively. The reduction caused by 5-ASA treatment was 67 and 69%, respectively.

## 4. Discussion

Our study shows that diethyl 3-nonyl-5-oxo-3,5,6,6a-tetrahydro-1*H*-cyclopenta[*c*]furan-4-ylphosphonate (P-5) (Scheme 1), a vinylphosphonate compound, containing a furan ring fused to a cyclopentenone ring, showed profound anti-inflammatory activity, in LPS-activated macrophage cells and in colitis-induced rat model. Cyclopentenone compounds were already tested with respect to IBD. For example, Cuzzocrea and coworkers showed that the reactive *α*,*β*-unsaturated carbonyl group, located in the cyclopentenone ring can ameliorate proinflammatory activity by reducing the activation of the nuclear factor kappa light chain enhancer of activated B (NF-*κ*B) cells [33]. The LC_50_ of P-5 was 15–20 *μ*M (Figure 1). Its IC_50_ in LPS-activated macrophages was calculated to be 6.1 *μ*M, a concentration in which P-5 was not cytotoxic. A lower concentration (5 *μ*M) also showed a profound reduction in TNF*α* levels. Because phosphonates are potential zinc-dependent metalloproteinase inhibitors [21, 34], we examined the possible inhibitory effect of P-5 on purified human recombinant TACE [35, 36] and found no effect (Figure 3).

Although TNF*α* is a major player in chronic inflammation and its amelioration [4, 37], other mediators, cytokines [38, 39], and chemokines [40–42] are involved in the inflammatory process. Consequently, the effect of P-5 in attenuating IL-6, IL-1*α*, INF-*γ*, MCP-1, MIP-1*α*, and RANTES in activated macrophages was tested in the concentration range of 1–20 *μ*M. The results shown in Figure 4 demonstrate that the compound possessed a profound anti-inflammatory effect, which did not involve TACE inhibition (Figure 3). An alternative mode of action could be an involvement in one or more signaling transduction pathways that are activated by LPS and/or the inflammation cascade. Indeed, the last part of the study explored the possible effect of P-5 on the activity of extracellular signal-regulated kinases (ERK) [43] and p38 [44] of the MAPK inflammation mediator family [45], as well as its possible involvement in the NF-*κ*B pathway [46, 47]. As demonstrated in Figure 5, P-5 did not prevent the degradation of I*κ*B*α*, which may indicate that P-5 did not affect NF-*κ*B. Also, P-5 did not interfere with the phosphorylation of p38; however, it did reduce the phosphorylation of ERK at two concentrations, 1 and 5 *μ*M.

In the last step of the study, the anti-inflammatory effect of P-5 was verified* in vivo* in the DNBS-induced rat model. A preliminary study was conducted in an attempt to identify the optimal effective dose in which P-5 reduces inflammation, when it is administrated intracolonically. The concentration was found to be 10 mg/kg body weight (data not shown). This dose of P-5 or 5-ASA (268 mg/kg body weight) as a positive control was administered rectally twice daily for three days. The macroscopic analysis shown in Table 1 demonstrates that the anti-inflammatory effect of P-5 was similar to that of 5-ASA. The biochemical analysis performed on the rat colon specimens confirmed the assumption that P-5 could serve as a local therapeutic agent in the treatment of IBD. In addition to reducing MPO activity to values similar to those in healthy colon, it reduced the mucosal levels of TNF*α* and IL-1*β* (Figure 6), a pattern akin to what was observed in the LPS-activated macrophages. P-5 administration also decreased iNOS expression in the treated colons, which could have led to reduced tissue NO levels. All this suggested that P-5 activity may involve amelioration of inflammation-driven oxidative stress [48]. This antioxidant activity of P-5 could result from its enone moiety in the cyclopentenone ring. The electrophilic nature of the *α*,*β*-unsaturated ketone can readily interact with reactive oxygen species, thus leading to termination of a free radical chain reaction occurring under oxidative conditions [49].

## 5. Conclusion

The fused-cyclopentenone phosphonate compound, P-5, possesses TACE-independent anti-inflammatory activity. Its mode of action involves reduction of the phosphorylation of ERK but it does not affect p38 or I*κ*B*α* and, hence, it has no effect on the expression of NF-*κ*B transcription factor. We speculate that P-5 may serve as an anti-inflammatory agent in the local treatment of colitis; however, human studies substantiation is required.

## Figures and Tables

**Table 1:** The effect of P-5 and 5-ASA on induced colitis, as assessed by measuring colon weight and ulceration scoring.

Group	Colon weight (mg/kg body weight)	Ulcer severity scoring
Healthy	4.3 ± 0.6	0
DNBS-induced, untreated	8.3 ± 1.9	1.9 ± 0.7
P-5 (10 mg/kg)	5.6 ± 0.5	0.2 ± 0.4
5-ASA (268 mg/kg)	4.7 ± 0.7	0.3 ± 0.4

Shown are the mean ± S.D. values of 5–10 rats.

## Abbreviations

DNBS: 2,4-Dinitrobenzene sulfonic acid
INF*γ*: Interferon *γ*
IBD: Inflammatory bowel disease
IL: Interleukin
iNOS: Inducible nitric oxide synthase
LPS: Lipopolysaccharide
MCP-1: Monocyte chemotactic protein-1
MIP-1*α*: Macrophage inflammatory protein 1*α*
MPO: Myeloperoxidase
RANTES: Regulated on activation normal T cell expressed and secreted
TACE: TNF*α*-converting enzyme
TNF*α*: Tumor necrosis factor alpha.

## References

[1] Monteleone G., Pallone F., MacDonald T. T. (2011). Emerging immunological targets in inflammatory bowel disease. *Current Opinion in Pharmacology*.

[2] O'Neill L. A. J. (2006). Targeting signal transduction as a strategy to treat inflammatory diseases. *Nature Reviews Drug Discovery*.

[3] Baumgart D. C., Sandborn W. J. (2007). Inflammatory bowel disease: clinical aspects and established and evolving therapies. *The Lancet*.

[4] Bradley J. R. (2008). TNF-mediated inflammatory disease. *Journal of Pathology*.

[5] Gupta G., Gelfand J. M., Lewis J. D. (2005). Increased risk for demyelinating diseases in patients with inflammatory bowel disease. *Gastroenterology*.

[6] Ljung T., Karlén P., Schmidt D. (2004). Infliximab in inflammatory bowel disease: clinical outcome in a population based cohort from Stockholm County. *Gut*.

[7] Hansen R. A., Gartlehner G., Powell G. E., Sandler R. S. (2007). Serious adverse events with infliximab: analysis of spontaneously reported adverse events. *Clinical Gastroenterology and Hepatology*.

[8] Bongartz T., Sutton A. J., Sweeting M. J., Buchan I., Matteson E. L., Montori V. (2006). Anti-TNF antibody therapy in rheumatoid arthritis and the risk of serious infections and malignancies: systematic review and meta-analysis of rare harmful effects in randomized controlled trials. *The Journal of the American Medical Association*.

[9] Naito Y., Yoshikawa T. (2005). Role of matrix metalloproteinases in inflammatory bowel disease. *Molecular Aspects of Medicine*.

[10] Gearing A. J. H., Beckett P., Christodoulou M. (1994). Processing of tumour necrosis factor-alpha precursor by metalloproteinases. *Nature*.

[11] Black R. A., Rauch C. T., Kozlosky C. J. (1997). A metalloproteinase disintegrin that releases tumour-necrosis factor-alpha from cells. *Nature*.

[12] Brynskov J., Foegh P., Pedersen G. (2002). Tumour necrosis factor *α* converting enzyme (TACE) activity in the colonic mucosa of patients with inflammatory bowel disease. *Gut*.

[13] Krishnaa A. B., Reddya M. V. N., Reddya G. C. S., Krishnaa B. S., Nayakb S. K., Reddy C. S. (2010). Synthesis, anti-oxidant and anti-bacterial properties of diethyl (4-flouro-3-nitro phenylamino) (substituted phenyl) methyl phosphonates. *The International Journal of Applied Biology and Pharmaceutical Technology*.

[14] Ternan N. G., Mc Grath J. W., Mc Mullan G., Quinn J. P. (1998). Organophosphonates: occurrence, synthesis and biodegradation by microorganisms. *World Journal of Microbiology and Biotechnology*.

[15] Stein C. A., Tonkinson J. L., Yakubov L. (1991). Phosphorothioate oligodeoxynucleotides—antisense inhibitors of gene expression?. *Pharmacology and Therapeutics*.

[16] Marshall L. A., Bolognese B., Yuan W., Gelb M. (1991). Phosphonate-phospholipid analogues inhibit human phospholipase A2. *Agents and Actions*.

[17] Boduszek B., Brown A. D., Powers J. C. (1994). Alpha-aminoalkylphosphonate di(chlorophenyl) esters as inhibitors of serine proteases. *Journal of Enzyme Inhibition*.

[18] Kiss T., Lázár I., Kafarski P. (1994). Chelating tendencies of bioactive aminophosphonates. *Metal-Based Drugs*.

[19] Agamennone M., Campestre C., Preziuso S. (2005). Synthesis and evaluation of new tripeptide phosphonate inhibitors of MMP-8 and MMP-2. *European Journal of Medicinal Chemistry*.

[20] Galezowska J., Gumienna-Kontecka E. (2012). Phosphonates, their complexes and bio-applications: a spectrum of surprising diversity. *Coordination Chemistry Reviews*.

[21] Pergament I., Reich R., Srebnik M. (2002). Novel matrix metallo-proteinase (MMP-2) phosphonoboronate inhibitors. *Bioorganic and Medicinal Chemistry Letters*.

[22] Quntar A. A. A. A., Gallily R., Katzavian G., Srebnik M. (2007). Potent anti-inflammatory activity of 3-aminovinylphosphonates as inhibitors of reactive oxygen intermediates, nitric oxides generation, and tumor necrosis factor-alpha release. *European Journal of Pharmacology*.

[23] Harel E., Rubinstein A., Chen W., Breuer E., Tirosh B. (2010). Aminoalkylcarbamoylphosphonates reduce TNFalpha release from activated immune cells. *Bioorganic and Medicinal Chemistry Letters*.

[24] Matsui T., Takahashi S., Matsunaga N. (2002). Discovery of novel phosphonic acid derivatives as new chemical leads for inhibitors of TNF-*α* production. *Bioorganic and Medicinal Chemistry*.

[25] Moradov D., Quntar A. A. A. A., Youssef M., Smoum R., Rubinstein A., Srebnik M. (2009). Mo(CO)6-mediated intramolecular Pauson-Khand reaction of substituted diethyl 3-allyloxy-1-propynylphosphonates. *Journal of Organic Chemistry*.

[26] Van Dyk D. E., Marchand P., Bruckner R. C. (1997). Comparison of snake venom reprolysin and matrix metalloproteinases as models of TNF-*α* converting enzyme. *Bioorganic and Medicinal Chemistry Letters*.

[27] Wallace J. L., Le T., Carter L., Appleyard C. B., Beck P. L. (1995). Hapten-induced chronic colitis in the rat: alternatives to trinitrobenzene sulfonic acid. *Journal of Pharmacological and Toxicological Methods*.

[28] Jubeh T. T., Nadler-Milbauer M., Barenholz Y., Rubinstein A. (2006). Local treatment of experimental colitis in the rat by negatively charged liposomes of catalase, TMN and SOD. *Journal of Drug Targeting*.

[29] Klotz U. (2000). The role of aminosalicylates at the beginning of the new millennium in the treatment of chronic inflammatory bowel disease. *European Journal of Clinical Pharmacology*.

[30] Storr M. A., Keenan C. M., Zhang H., Patel K. D., Makriyannis A., Sharkey K. A. (2009). Activation of the cannabinoid 2 receptor (CB2) protects against experimental colitis. *Inflammatory Bowel Diseases*.

[31] Grisham M. B., Benoit J. N., Granger D. N. (1990). Assessment of leukocyte involvement during ischemia and reperfusion of intestine. *Methods in Enzymology*.

[32] Bradford M. M. (1976). A rapid and sensitive method for the quantitation of microgram quantities of protein utilizing the principle of protein dye binding. *Analytical Biochemistry*.

[33] Cuzzocrea S., Ianaro A., Wayman N. S. (2003). The cyclopentenone prostaglandin 15-deoxy-Δ^12,14^- PGJ_2_ attenuates the development of colon injury caused by dinitrobenzene sulphonic acid in the rat. *British Journal of Pharmacology*.

[34] Farkas E., Katz Y., Bhusare S. (2004). Carbamoylphosphonate-based matrix metalloproteinase inhibitor metal complexes: solution studies and stability constants. Towards a zinc-selective binding group. *Journal of Biological Inorganic Chemistry*.

[35] Black R. A. (2002). Tumor necrosis factor-alpha converting enzyme. *International Journal of Biochemistry and Cell Biology*.

[36] Moss M. L., Jin S.-L. C., Becherer J. D. (1997). Structural features and biochemical properties of TNF-*α* converting enzyme (TACE). *Journal of Neuroimmunology*.

[37] Di Girolamo N., Visvanathan K., Lloyd A., Wakefield D. (1997). Expression of TNF-*α* by human plasma cells in chronic inflammation. *Journal of Leukocyte Biology*.

[38] Sanchez-Muñoz F., Dominguez-Lopez A., Yamamoto-Furusho J. K. (2008). Role of cytokines in inflammatory bowel disease. *World Journal of Gastroenterology*.

[39] Strober W., Fuss I. J. (2011). Proinflammatory cytokines in the pathogenesis of inflammatory bowel diseases. *Gastroenterology*.

[40] MacDermott R. P. (1999). Chemokines in the inflammatory bowel diseases. *Journal of Clinical Immunology*.

[41] Nishimura M., Kuboi Y., Muramoto K., Kawano T., Imai T. (2009). Chemokines as novel therapeutic targets for inflammatory bowel disease. *Annals of the New York Academy of Sciences*.

[42] McCormack G., Moriarty D., O'Donoghue D. P., McCormick P. A., Sheahan K., Baird A. W. (2001). Tissue cytokine and chemokine expression in inflammatory bowel disease. *Inflammation Research*.

[43] Liew C. Y., Lam K. W., Kim M. K. (2011). Effects of 3-(2-Hydroxyphenyl)-1-(5-methyl-furan-2-y-l) propenone (HMP) upon signalling pathways of lipopolysaccharide-induced iNOS synthesis in RAW 264.7 cells. *International Immunopharmacology*.

[44] Pargellis C., Tong L., Churchill L. (2002). Inhibition of p38 MAP kinase by utilizing a novel allosteric binding site. *Nature Structural Biology*.

[45] Kaminska B. (2005). MAPK signalling pathways as molecular targets for anti-inflammatory therapy—from molecular mechanisms to therapeutic benefits. *Biochimica et Biophysica Acta*.

[46] Lee J. Y., Jhun B. S., Oh Y. T. (2006). Activation of adenosine A_3_ receptor suppresses lipopolysaccharide-induced TNF-*α* production through inhibition of PI 3-kinase/Akt and NF-*κ*B activation in murine BV2 microglial cells. *Neuroscience Letters*.

[47] Straus D. S., Pascual G., Li M. (2000). 15-deoxy-delta 12,14-prostaglandin J2 inhibits multiple steps in the NF-kappa B signaling pathway. *Proceedings of the National Academy of Sciences*.

[48] Grisham M. B., Granger D. N. (1988). Neutrophil-mediated mucosal injury: role of reactive oxygen metabolites. *Digestive Diseases and Sciences*.

[49] Shang Y.-J., Jin X.-L., Shang X.-L. (2010). Antioxidant capacity of curcumin-directed analogues: structure-activity relationship and influence of microenvironment. *Food Chemistry*.

